# Effects of macro- versus nanoporous silicon substrates on human aortic endothelial cell behavior

**DOI:** 10.1186/1556-276X-9-421

**Published:** 2014-08-21

**Authors:** Pilar Formentín, María Alba, Úrsula Catalán, Sara Fernández-Castillejo, Josep Pallarès, Rosà Solà, Lluís F Marsal

**Affiliations:** 1 Nano-electronic and Photonic Systems, Departament d’Enginyeria Electrònica, Elèctrica I Autómatica, Universitat Rovira i Virgili, Països Catalans 26, Tarragona 43007, Spain; 2 Unit of Lipids and Atherosclerosis Research, Facultat de Medicina I Ciències de la Salut, Universitat Rovira i Virgili, Sant Llorenç 21, Reus, Tarragona 43201, Spain

**Keywords:** Porous silicon, HAEC, Cell adhesion, Cell morphology

## Abstract

Human aortic endothelial cells play a key role in the pathogenesis of atherosclerosis, which is a common, progressive, and multifactorial disease that is the clinical endpoint of an inflammatory process and endothelial dysfunction. Study and development of new therapies against cardiovascular disease must be tested in vitro cell models, prior to be evaluated in vivo*.* To this aim, new cell culture platforms are developed that allow cells to grow and respond to their environment in a realistic manner. In this work, the cell adhesion and morphology of endothelial cells are investigated on functionalized porous silicon substrates with two different pore size configurations: macroporous and nanoporous silicon. Herein, we modified the surfaces of porous silicon substrates by aminopropyl triethoxysilane, and we studied how different pore geometries induced different cellular response in the cell morphology and adhesion. The cell growth over the surface of porous silicon becomes an attractive field, especially for medical applications. Surface properties of the biomaterial are associated with cell adhesion and as well as, with proliferation, migration and differentiation.

## Background

Human aortic endothelial cells (HAECs) have been the most commonly used model in endothelial dysfunction systems. The endothelium serves as a natural barrier to prevent platelet adhesion and thrombosis. Disruption of the endothelium can lead to thrombosis, inflammation, and restenosis. Although drug-eluting stents are employed to minimize restenosis, there are reports of late thrombosis associated with the use of these drugs. It is believed that these effects are due to the slow growth of the endothelial cells to regenerate the endothelium monolayer of the stent material [1]. Because of the capacity of these cells to adhere to the substrate and to produce cell adhesion molecules, HAECs seem to be a good cell model to screen new cardiovascular therapies. Surface modifications have been applied to improve cell adhesion and accelerated cell growth on biomaterials [2]. Improvements on the surface of biomaterials are needed, particularly for endothelial cells, which exhibit poor adhesion and slow growth on biomaterials.

The properties of porous silicon (pSi) make it an interesting material for biological application. PSi is biodegradable, and it dissolves into nontoxic silicic acid. This behavior depends on the properties of the porous layer [3-5]. The pore diameter can be controlled, and a variety of pore sizes can be produced by changing the etching conditions [6-8]; also, the high surface area can be loaded with a range of bioactive species. For all this, pSi has been proposed and used for in vitro and in vivo biological applications [9-14]. Substrate topography affects cell functions, such as adhesion, proliferation, migration, and differentiation [15-17], and the influence of the pore size on the proliferation and morphology of cells adhered has been studied [18,19].

A variety of surface functional groups have been evaluated to improve cell adhesion and growth, such as amines, imines, esters, or carboxylic acids [20-22]. The most common and simple surface treatment is oxidation, which can be performed by either ozone, aging, thermal, or chemical treatments. Amine-terminated modifications as silanization with aminopropyl triethoxysilane or triethoxysilane improve pSi stability and enhance cell adhesion in comparison to oxidized pSi [9].

Herein, we report the cell adhesion and cell morphology of HAEC on macro- and nanoporous silicon substrates silanized with aminopropyl triethoxysilane (APTES). PSi substrates were fabricated by electrochemical etching of silicon wafers in a hydrofluoric acid (HF) solution. Macro- and nanopore configurations were achieved changing the Si substrate, the electrolyte content, and the current density [23-25]. The samples were surface-modified by oxidation and silanization with APTES [26] in order to improve surface stability and to promote cell adhesion and proliferation. The interactions between cells and Si substrates have been characterized by confocal and scanning electron microscopy (SEM), and the results show the effect of the surface topography on the HAEC behavior compared to the flat silicon. This study demonstrates potential applications of these forms of silicon for controlling cell development in tissue engineering as well as in basic cell biology research.

## Methods

### Porous silicon fabrication

P-type <100 > silicon wafers with a resistivity of 0.002 to 0.004 Ω cm were used for etching nanoporous silicon (NanPSi). Silicon wafers with a resistivity of 10 to 20 Ω cm were used for macroporous silicon (MacPSi). All pSi were prepared using an anodization process in a custom-made Teflon etching cell. An electrolyte formed by combining hydrofluoric acid (HF 48%) with ethanol and glycerol with the ratio of 3:7:1 (*v*/*v*), respectively, was used for the anodization of NanPSi, and an electrolyte of hydrofluoric acid (40%) in *N*,*N*-dimethylformamide (DMF) (1:10) was made for MacPSi etching. For NanPSi, the wafer was etched with a current density of 60 mA/cm^2^ for 1 min. MacPSi was etched with a current density of 4 mA/cm^2^ for 30 min. Then, the samples were rinsed with pentane and dried under a nitrogen flow. Macro- and nanoporous silicon samples were morphologically characterized by scanning electron microscopy (ESEM-FEI Quanta 600 and SEM Quanta 450; FEI, Hillsboro, OR, USA).

### Porous silicon functionalization

MacPSi and NanPSi substrates were oxidized at 600°C for 15 min. Then, the samples were treated in KOH 0.1 M for 3 min and HNO_3_ 0.1 M for 10 min to increase the density of surface hydroxyl groups. Next, the samples were silanized in 5 mM solution of APTES (Gelest Inc., Morrisville, PA, USA) in anhydrous toluene for 3 h at 75°C. Then, they were washed in succession with toluene, ethanol, and deionized water and dried under a nitrogen flow.

### Cell seeding and culture

HAECs were purchased from Cascade Biologics^TM^ (Portland, OR, USA) and, at the 5th passage, were thawed and seeded on Nunclon^TM^ Δ surface 12-well plates (Thermo Fisher Scientific, Waltham, MA, USA) in the presence or absence (in the case of control conditions) of sterilized silicon substrates, at a density of approximately 1.9 × 10^4^ viable cells/mL and 4 × 10^3^ of viable cells/cm^2^. Through the whole experiment, cells were maintained in M200 medium supplemented with 2% (*v*/*v*) low serum growth supplement (LSGS), 10 mg/mL gentamicin, 0.25 mg/mL amphotericin B, 100 U/mL penicillin, and 100 mg/mL of streptomycin.

Cells were seeded in complete cell culture medium and growth at 37°C in a humidified incubator (HERAcell 150; Heraeus, Hanau, Denmark) with atmosphere containing 5% CO_2_, and culture medium was replenished every 2 days with a fresh medium.

### Cell viability and cytotoxicity

Cell viability was assessed by morphology using phase-contrast microscopy and by trypan blue exclusion (Merck & Co., Inc., Whitehouse Station, NJ, USA). The viability of the HAEC was >97%.

The extent of cytotoxicity of each experimental condition was determined by a colorimetric assay, which measures released lactate dehydrogenase (LDH) activity (the LDH Cytotoxicity Detection Kit; Roche Applied Science, Penzberg, Germany). Briefly, LDH enzyme is rapidly released into the cell culture supernatant when the plasma membrane is damaged. This result is a colorimetric reaction that can be measured at a wavelength of 492 nm. Thus, the activity of LDH released by the cells was measured in cell-free supernatants collected after 48-h incubation times. Results are expressed as mean 492-optical density (OD) and standard deviation (SD error bars) of LDH produced by the cells under each treatment condition.

### Scanning electron microscopy

The morphology and shape of cells adhering to the functionalized PSi substrates were observed with scanning electron microscope (SEM) (JEOL model JSM-6400; JEOL Ltd., Akishima-shi, Japan). The adhered HAECs were fixed in a fixative containing 2.5% GTA/0.1 M phosphate buffered saline (PBS) at room temperature for 2 h. After washing twice with 0.1 M PBS, the cells were postfixed with 1% osmium tetroxide at temperature for 1 h. The cells were then washed twice with PBS, dehydrated through serial gradients of ethanol (10 min per each gradient), and finally dried out by the critical point dryer Bal-Tec CPD-030 (Bal-Tec AG, Balzers, Liechtenstein). The cells along with the substrates were sputtered with gold at a current of 15 mA for 3 min by the ion sputter EMITECH K575X. SEM imaging was conducted at voltages ranging from 5 to 10 kV.

### Staining on actin and nuclei and fluorescence confocal microscopy

HAECs were cultured on the functionalized pSi substrates for 48 h. After cell culture experiments, culture media were removed and cells were washed two times with PBS at 37°C. The cells were fixed with a 4% (*w*/*v*) solution of paraformaldehyde in PBS for 30 min at room temperature. After washing two times more with PBS, the substrates were immersed in 0.2% Triton-X 100 in PBS for 10 min at room temperature to permeabilize the cell membrane. After rinsing with PBS two times, the actin filaments and nuclei were stained in the dark at room temperature. Actin-stain 670 phalloidin (tebu-bio, Le Perray-en-Yvelines, France) was used to stain the actin filaments (200 nM, 30 min), while NucGreen Dead 488 (Life Technologies, Carlsbad, CA, USA) was used to stain the nuclei (two drops/mL, 10 min). Each sample was washed three times with PBS, and after mounting on microscope slides using anti-fade mounting media, the samples were incubated overnight in the dark at room temperature. Stained cells were kept at 4°C in the dark until microscope observations. The fluorescence images were acquired using a Nikon Eclipse TE2000-E inverted microscope (Nikon Instruments, Amsterdam, Netherlands), equipped with a C1 laser confocal system (EZ-C1 software, Nikon). Argon 488- and 633-nm lasers were used as excitation sources for NucGreen and phalloidin, respectively.

## Results and discussion

**Figure 1:**
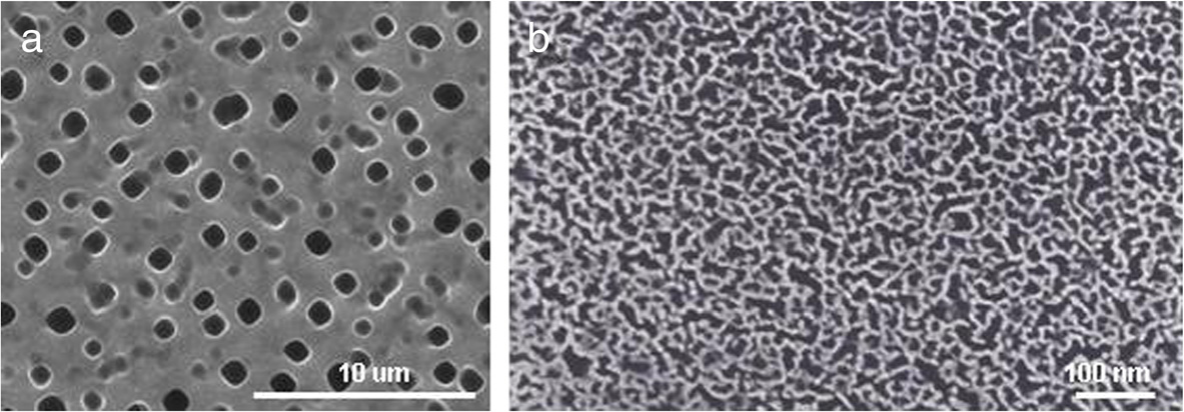
**Morphological characterization of porous silicon substrates.** Top view ESEM images of **(a)** macroporous silicon substrate with a pore diameter of 1 to 1.5 μm and **(b)** nanoporous silicon with pore sizes less than 50 nm.

**Figure 2:**
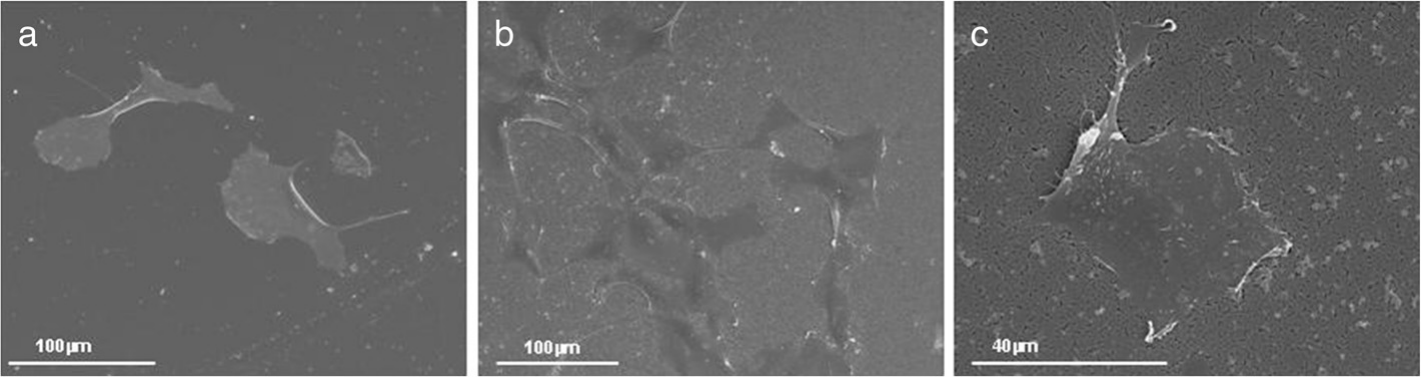
**SEM characterization of endothelial cells on nanoporous silicon.** SEM images of HAEC culture after 48-h incubation on modified silicon substrates: **(a)** flat silicon and **(b, c)** nanoporous silicon.

**Figure 3:**
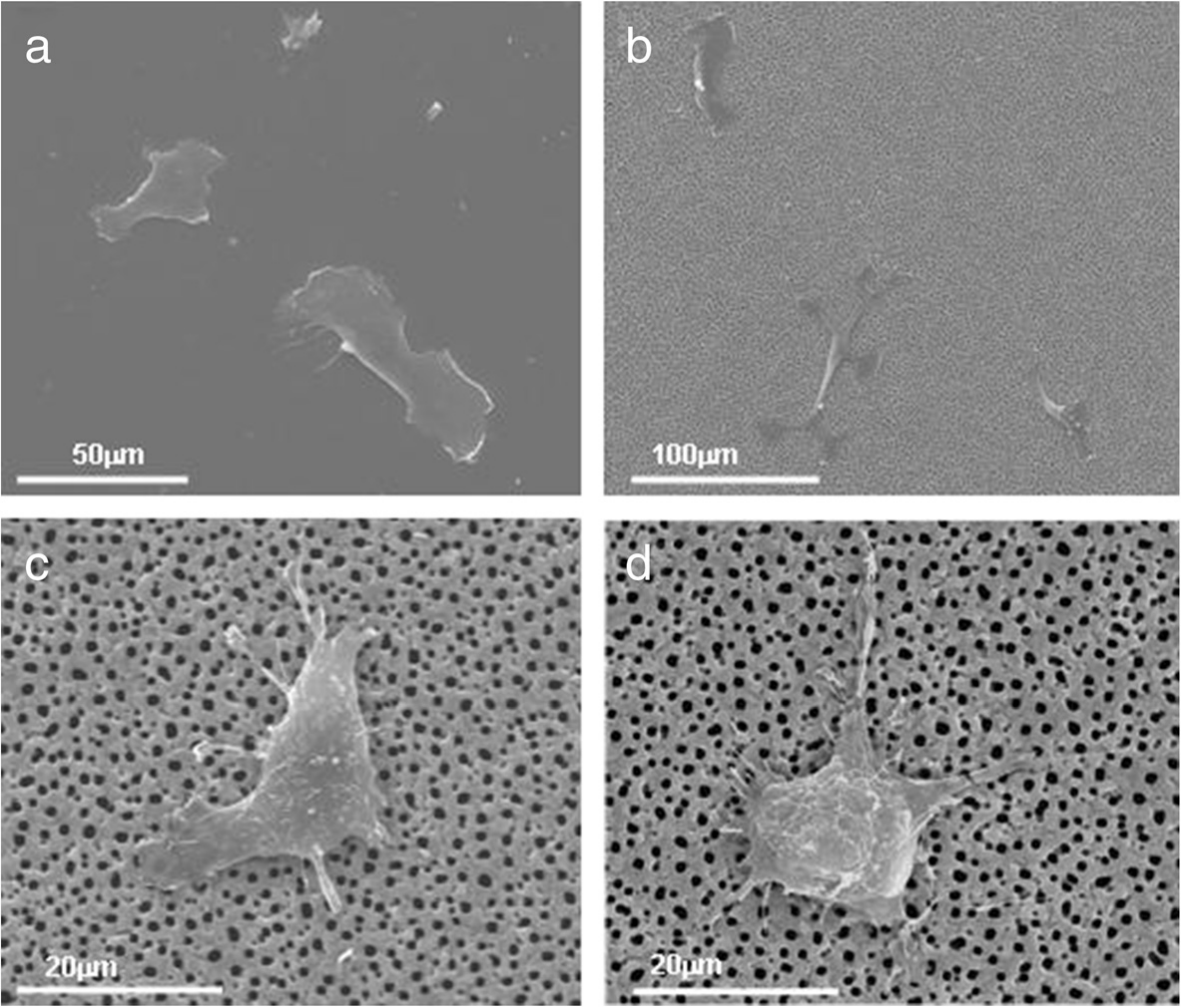
**SEM characterization of HAECs on macroporous silicon.** SEM images of HAEC culture after 48-h incubation on modified silicon substrates: **(a)** flat silicon and **(b, c, d)** macroporous silicon substrates.

**Figure 4:**
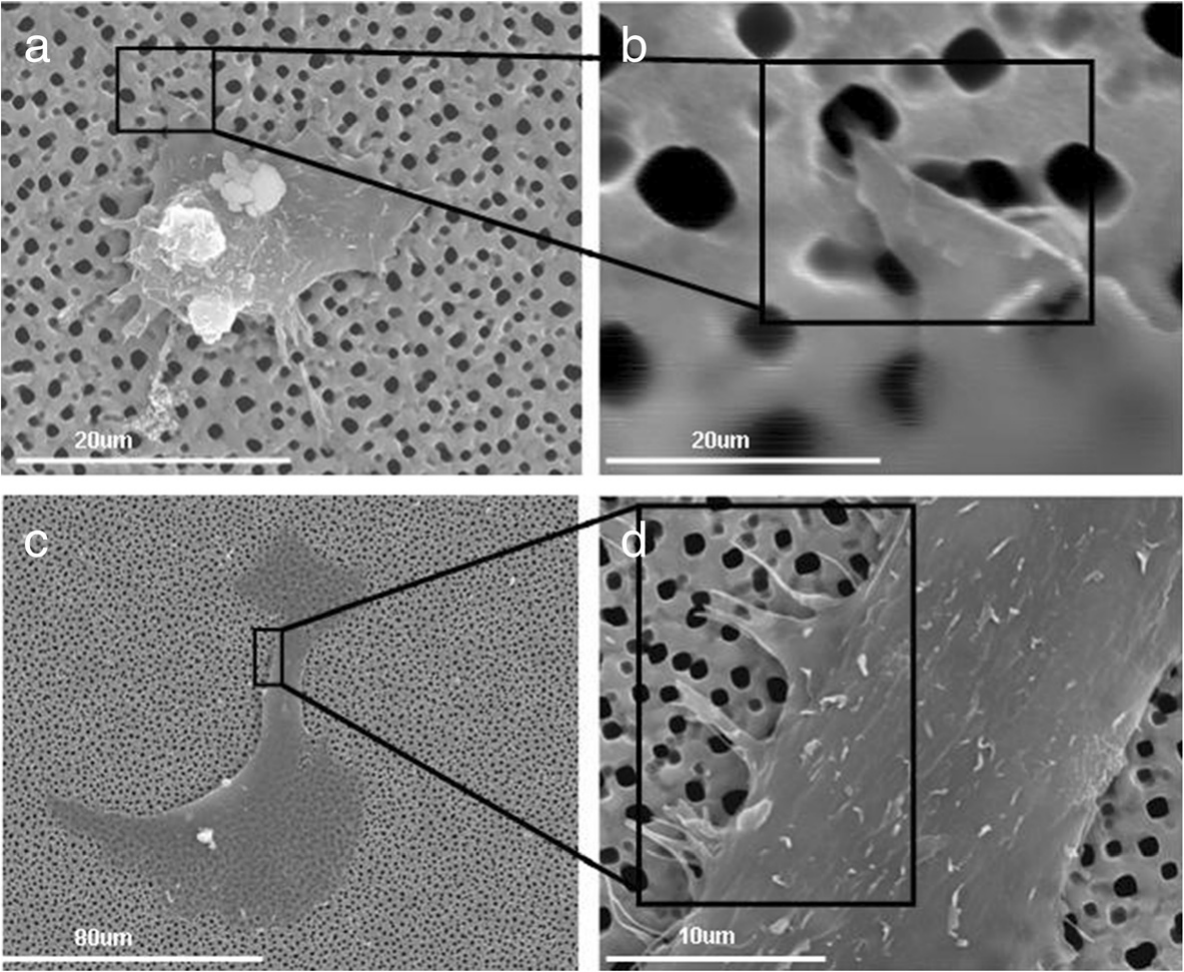
**Images of HAECs growing on macroporous silicon substrates. (a, b, c, d)** SEM images of HAEC culture after 48-h incubation on modified macroporous silicon at different magnification.

**Figure 5:**
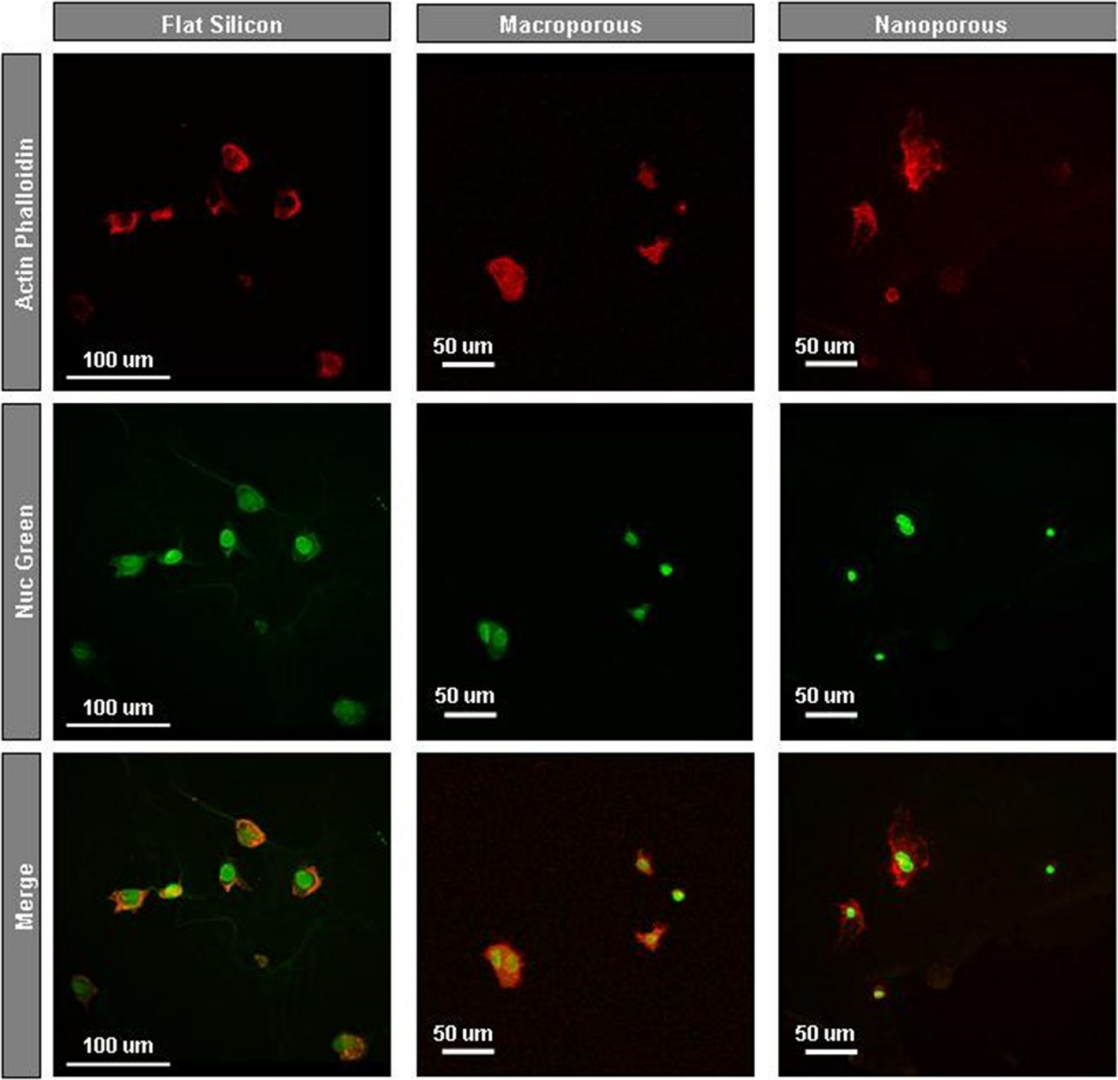
**Fluorescence confocal microscopy.** Confocal imaging for HAECs cultured on three different substrates at 37°C for 48-h incubation. The actin filaments were stained with actin-stain 670 phalloidin for 30 min (red), and the nucleus was stained with NucGreen Dead 488 for 10 min (green).

The porous silicon (pSi) samples were produced by electrochemical etching of p-type silicon wafers in HF-based electrolytes [22]. Two types of samples were generated by varying the etching conditions in order to study the cellular response on surfaces with different pore geometry. PSi substrates obtained from silicon wafers with a resistivity of 0.002 to 0.004 Ω cm by applying a constant current density of 60 mA/cm^2^ had an average pore diameter of 30 to 50 nm. The pSi produced from silicon wafers with 10 to 20 Ω cm resistivity, by applying a current density of 4 mA/cm^2^, had an average pore diameter of 1 to 1.5 μm.The topography of theses substrates was analyzed using scanning electron microscopy. Figure 1a,b shows representative images of the top view of macro- and nanoporous substrates, which were surface-modified by oxidation and silanization with APTES to promote cell adhesion.Human endothelial cell line was chose to analyze the influence of the topography of the macro- and nanoporous silicon on the HAEC behavior. To study the effect of the pore size on the morphology of the adhered HAECs, confocal microscopy and SEM were employed. Figure 2 shows representative images of HAECs growing on nanoporous Si substrate and on flat Si as control, after 48 h of incubation. On porous silicon, cells appeared elongated and spread with protrusions, and the development of the filopodia is visible at the cell borders (Figure 2b,c), which is because the nanopores may not anchor firmly to the surface. The same shape is observed on flat silicon (Figure 2a).Figures 3 and 4 illustrate the results obtained on macroporous silicon substrates. These indicate the effect of the surface in the cell adhesion and spreading, compared to the flat Si. The cell migration after 48-h incubation on pSi 1 to 1.5 μm results in 2-D and 3-D shape of the HAEC, while the cells on nano and flat silicon show only 2-D migration movements. In the macroporous substrate, the cell appears with a well-spread cytoskeleton with formation of protrusions out of the cell membrane and is visible how part of it penetrates inside the macropore (Figure 4b,d). Filopodia is not present in this type of substrate.Figure 5 shows confocal imaging for HAEC culture on flat, macro-, and nanoporous silicon modified with APTES. The samples were washed after 48 h of incubation, and then, the remaining cells were fixed and labeled with actin phalloidin and NucGreen.

From fluorescence microscopy, we notice that the fluorescence images provided limited information on cell morphology to qualify the cell development on these three types of silicon substrates. On flat silicon, the cell looks more spread over the substrate (flat shape). On the two types of porous silicon, the spreading behaviors of the cells look limited by the topography of the surface.

Cytotoxicity was determined by a colorimetric assay, which measures released LDH activity. LDH enzyme is released into the cell culture when the membrane is damaged. So, an increase of LDH has been associated with a cellular injury. After a period of 48 h, the production of LDH activity released increases in the porous silicon substrates and also in the blank control (cells incubated without silicon substrates). These results indicate that the presence of the silicon in the culture medium does not cause cytotoxicity *per se*.

To quantify viability of cells grown on surface porous silicon, we assessed the morphology using phase-contrast microscopy and by trypan blue exclusion (Merck & Co., Inc.).

The cell viability of HAECs was >97% in all the porous substrates.

## Conclusions

Silicon substrates with pore size in the macro- and nanoporous range have been used to study the adhesion and the morphology of endothelial cells. The substrates were functionalized previously, with APTES in order to improve the adhesion.

SEM characterization shows that different pore geometries induced different cellular response in terms of cell adhesion and morphology. On macroporous silicon, the pseudopods of the cell can grow along the macropore, and the cells show 2-D and 3-D migration behaviors. On nanoporous substrates, filopodia was found to branch out from the main cell body, which anchors the cell to the substrate.

From fluorescence microscopy, limited information on cell morphology to qualify the cell development on these silicon substrates is obtained.

These two forms of porous silicon, macro and nano, are promising substrates for developing new 3-D cell culture platforms with applications in tissue engineering as well as basic cell biology research.

## Abbreviations

HAEC: human aortic endothelial cells; pSi: porous silicon; APTES: aminopropyl triethoxysilane; MacPSi: macroporous silicon; NanPSi: nanoporous silicon; HF: hydrofluoric acid; DMF: *N, N* dimethylformamide; KOH: potassium hydroxide; HNO_3_: nitric acid; LDH: lactate dehydrogenase; GTA: glutaraldehyde; PBS: phosphate buffered saline; OD: optical density; SD: standard deviation; SEM: scanning electron microscopy; ESEM: environmental scanning electron microscopy.

## Competing interests

The authors declare that they have no competing interests.

## Authors’ contributions

The work presented here was carried out in collaboration among all authors. The experiments presented in this work were designed by PF and LFM. The pSi substrates were fabricated and functionalized by MA and characterized microscopically by PF and MA. Cell seeding and culture, cell viability, and cytotoxicity were carried out by UC, SFC, and RS. SEM characterization after 48 h-incubation was analyzed by PF. MA, PF, UC, SFC, JP, RS, and LFM analyzed and discussed the results obtained from the experiments. PF wrote the manuscript, and it was revised by all the authors (PF, MA, UC, SFC, JP, RS, and LFM). All authors read and approved the final manuscript.

## References

[B1] Bhattacharyya D Xu H Deshmukh RR Timmons RB Nguyen KT Surface chemistry and polymer film thickness effects on endothelial cell adhesion and proliferation J Biomed Mater Res A 2010 9 640 648 20213813 10.1002/jbm.a.32713 PMC2892191

[B2] Kasemo B Biological surface science Surf Sci 2002 9 656 677 10.1016/S0039-6028(01)01809-X

[B3] Anderson SHC Elliot H Wallis DJ Canham LT Powell JJ Dissolution of different forms of partially porous silicon wafers under simulated physiological conditions Phys Status Solid A 2003 9 331 335

[B4] Park JH Gu L von Maltzahn G Ruoslahti E Bhatia SN Sailor MJ Biodegradable luminescent porous silicon nanoparticles for in vivo applications Nat Mater 2009 9 331 336 10.1038/nmat2398 19234444 PMC3058936

[B5] Canham LT Bioactive silicon structure fabrication through nanoetching techniques Adv Mater 1995 9 1033 1037 10.1002/adma.19950071215

[B6] Canham LT Eds Porosity Properties of Porous Silicon 1997 London: Institution of Engineering and Technology 416

[B7] Janshoff A Dancil KPS Steinem C Greiner DP Lin VSY Gurtner C Motesharei K Sailor MJ Ghadiri MR Macroporous p-type silicon Fabry-Perot layers. Fabrication, characterizations and applications in biosensing J Am Chem Soc 1998 9 12108 12116 10.1021/ja9826237

[B8] Steward MP Buriak JM Chemical and biological applications of porous silicon technology Adv Mater 2000 9 859 869 10.1002/1521-4095(200006)12:12<859::AID-ADMA859>3.0.CO;2-0

[B9] Low SP Williams KA Canham LT Voelcker NH Evaluation of mammalian cell adhesion on surface-modified porous silicon Biomaterials 2006 9 4538 4546 10.1016/j.biomaterials.2006.04.015 16707158

[B10] Low SP Voelcker NH Canham LT Williams KA The biocompatibility of porous silicon in tissues of the eye Biomaterials 2009 9 2873 2880 10.1016/j.biomaterials.2009.02.008 19251317

[B11] Gentile F La Rocca R Marinaro G Nicastri A Toma A Paonessa F Cojoc G Liberale C Benfenati F di Fabrizio E Decuzzi P Differential cell adhesion on mesoporous silicon substrates ACS Appl Mater Interfaces 2012 9 2903 2911 10.1021/am300519a 22583790

[B12] Sweetman MJ Ronci M Ghaemi SR Craig JE Voelcker NH Porous silicon films micropatterned with bioelements as supports for mammalian cells Adv Funct Mater 2012 9 1158 1166 10.1002/adfm.201102000

[B13] Punzón-Quijorna E Sánchez-Vaquero V Muñoz-Noval A Pérez-Roldán MJ Martín-Palma R Rossi F Climent-Font A Manso-Silván M García-Ruiz JP Torres-Costa V Nanostructures porous silicon micropatterns as a tool for substrate-conditioned cell research Nanoscale Res Lett 2012 9 396 10.1186/1556-276X-7-396 22799489 PMC3458952

[B14] Hajdu K Gergely C Martin M Zimányi L Agarwal V Palestino G Hernádi K Németh Z Nagy L Light-harvesting bio-nanomaterial using porous silicon and photosynthetic reaction center Nanoscale Res Lett 2012 9 400 10.1186/1556-276X-7-400 22804837 PMC3442959

[B15] Curtis A Wilkinson C Topographical control of cells Biomaterials 1997 9 1573 1583 10.1016/S0142-9612(97)00144-0 9613804

[B16] Sun W Puzas JE Sheu TJ Liu X Fauchet PM Nano- to microscale porous silicon as a cell interface for bone-tissue engineering Adv Mater 2007 9 921 924 10.1002/adma.200600319

[B17] Dalby MJ Gadegaard N Tare R Andar A Riehle MO Herzyk P Wilkinson CDW Oreffo ROC The control of human mesenchymal cell differentiation using nanoscale symmetry and disorder Nat Mater 2007 9 997 1003.003 10.1038/nmat2013 17891143

[B18] Khung YL Barritt G Voelcker NH Using continuous porous silicon gradients to study the influence of surface topography on the behaviour of neuroblastoma cells Exp Cell Res 2008 9 789 800 10.1016/j.yexcr.2007.10.015 18054914

[B19] Collart-Dutilleu PY Secret E Panayootov I Deville de Périère D Martín-Palma R Torres-Costa V Martin M Gergely C Durand JO Cunin F Cuisinier FJ Adhesion and proliferation of human mesenchymal stem cells from dental pulp on porous silicon scaffolds ACS Appl Mater Interfaces 2014 9 1719 1728 10.1021/am4046316 24428409

[B20] Gumpenberger T Heitz J Bäuerle D Kahr H Graz I Romanin C Svorcik V Leisch F Adhesion and proliferation of human endothelial cells on photochemically modified polytetrafluoroethylene Bimaterials 2003 9 5139 5144 10.1016/S0142-9612(03)00460-5 14568430

[B21] Lakard S Herlem G Proper A Kastner A Michel G Vallès-Villarreal N Gharbi T Fahys B Adhesion and proliferation of cells on new polymers modified biomaterials Bioelectrochemistry 2004 9 19 27 10.1016/j.bioelechem.2003.09.009 14990322

[B22] Bisson I Kosinki M Ruault S Gupta B Hilborn J Wurm F Frey P Acrylic acid grafting and collagen immobilization on poly(ethylene terephthalate) surfaces for adherence and growth of human bladder smooth muscle cells Biomaterials 2002 9 3149 3158 10.1016/S0142-9612(02)00061-3 12102186

[B23] Trifonov T Marsal LF Rodríguez A Pallarès J Alcubilla R Fabrication of two- and three-dimensional photonic crystals by electrochemical etching of silicon Phys Status Solid C 2005 9 3104 3107

[B24] Trifonov T Rodríguez A Marsal LF Pallarès J Alcubilla R Macroporous silicon: a versatile material for 3D structure fabrication Sensors Actuators A 2008 9 662 669 10.1016/j.sna.2007.09.001

[B25] Xifré-Pérez E Marsal LF Ferré-Borrull J Low refractive index contrast porous silicon omnidirectional reflectors Appl Phys B 2009 9 169 172 10.1007/s00340-009-3416-0

[B26] Alba M Romano E Formentín P Eravuchira PJ Ferré-Borrull J Pallarès J Marsal LF Selective dual-side functionalization of hollow SiO_2_ micropillar arrays for biotechnological applications RSC Advances 2014 9 11409 11416 10.1039/c3ra48062c

